# Health orientation and individual tendencies of a sample of Italian genetic testing consumers

**DOI:** 10.1002/mgg3.1291

**Published:** 2020-06-05

**Authors:** Serena Oliveri, Ilaria Durosini, Ilaria Cutica, Clizia Cincidda, Francesca Spinella, Marina Baldi, Alessandra Gorini, Gabriella Pravettoni

**Affiliations:** ^1^ Department of Oncology and Hemato‐Oncology (DIPO) University of Milan Milan Italy; ^2^ Applied Research Division for Cognitive and Psychological Science IEO European Institute of Oncology IRCCS Milan Italy; ^3^ GENOMA Group Molecular Genetics Laboratories Rome Italy

**Keywords:** decision‐making, genetic testing, health orientation, public health, risk propensity

## Abstract

**Background:**

Over the last decade, genetic testing (GT) had markedly spread in European countries and struggled the debate concerning the psychological effects on the population. The aim of this study was to investigate the individual tendencies of GT consumers in a sample of Italian citizens.

**Methods:**

A total of 152 Italian clients from GenomaLab, a private genetic company, were enrolled from February 2016 to September 2018 and completed an ad hoc survey.

**Results:**

Results showed that GT consumers were motivated to preserve their well‐being, they felt responsible for their health, they were neither pessimistic nor optimistic toward negative occurrences, and poorly inclined to take high risks in their lives. Participants who had suffered from a disease in the past appear to be less tolerant to the uncertainty for future negative events.

**Conclusion:**

Our results depict Italian GT consumers as health‐oriented, focused on prevention, who do not have a pessimistic perception of their condition but do not like to “bet” on their health, and probably their intention (and belief) is to acquire genetic information in order to reduce uncertainty and increase their decision‐making “power” related to their health. Taken together, all these results contribute to describe the population of GT users in European countries, to regulate the provision of GT results and to entail the communication of genetic risk information based on a consumers’ personal profile.

## Graphical abstract

Italian GT consumers are health‐oriented, focused on prevention, do not have a pessimistic perception of their condition but do not like to "bet" on their health. Their main intention (and belief) is to acquire genetic information in order to reduce uncertainty and increase their decision‐making “power” on their health.

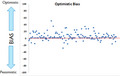

## 1 INTRODUCTION

Over the last decades, genetic testing (GT) has succeeded in the European market (Global Market Insight Inc, 19 June 2019) and people have been going to learn more about their health than they could manage. Some startups, such as *Color Genomics and Counsyl*, focus on revealing genetic risk for several kinds of genetic diseases. Other companies have created a DNA app marketplace, where health companies can use members’ data to offer a huge number of “product” suggestions. Additionally, the holy grail of genetic sequencing—mapping your whole genome—is slowly becoming more available to a large crowd of people (Crow, 2019; Grishin et al., 2018). The interesting aspect is that by learning their risk for certain health conditions, people can make an effort to prevent them, for instance, they can eat better, take the right medication, get involved in physical trainings, or get screened by doctors more often.

Several people may be really motivated to discover whether they are predisposed to be lactose or gluten intolerant, or if their metabolism is better than the average person, precisely because these conditions are manageable with changes in habits and/or medication intake (Fallaize, Macready, Butler, Ellis, & Lovegrove, 2013; Stewart‐Knox et al., 2008). Nevertheless, they might be much more worried or anxious about learning more serious issues, such as being more likely to get cancer or Alzheimer, without having a clear idea of what it is then possible to actively do with the genetic information received (Collins, Ryan, & Truby, 2014; Oliveri, Ferrari, Manfrinati, & Pravettoni, 2018; Oliveri, Pravettoni, Pravettoni, Fioretti, & Hansson, 2016). The debate between experts worried that consumers might be psychologically unprepared to handle frightening health information lasted for a long time (Oliveri & Pravettoni, 2016). It is well‐known how emotions impact the consumer decision‐making process, and how consumers are influenced by emotions in taking health decisions (Achar, So, Agrawal, & Duhachek, 2016). Additionally, life experiences and family history of illness can play a huge role in handling with genetic risk information and their implications (Oliveri, Renzi, Masiero, & Pravettoni, 2015), as does patients’ current context of life (e.g., knowing that a gene related to a specific disease runs in the family could affect procreative choices, or parenthood could affect the importance given to health prevention).

A recent review highlighted that people tend to be psychologically unprepared to receive genetic bad news, that there is a huge difference in the motivations which could guide an individual to undergo a genetic test and a huge difference in reactions after results based on the category of disease investigated by the test (Oliveri et al., 2018). Oliveri and colleagues (2018) described that the psychological distress due to genetic test results is often related to a full‐blown clinical condition or clinical symptoms which already affect the individual, rather than to the result itself. Based on the data in the literature, the authors highlighted that genetic risk for cardiovascular disease is perceived to be manageable, whereas neurodegenerative diseases (e.g., Alzheimer's disease, AD) or cancer (e.g., breast cancer, BC) could be addressed in advance with preventive choices (such as drug therapies for AD and prophylactic mastectomy for BC). Overall, people maintain confidence in being able to cope with their risk and tend to consider genetic tests as valid information to take important preventive decisions (Oliveri et al., 2018). Another study showed that health concern and curiosity often beats out the anxiety for genetic breaking bad news: despite people were uncomfortable about receiving results for conditions that had no treatment, around 61% of them wanted to know all of their whole genome sequencing results (Jamal et al., 2017). The choice of knowing about their genetic risk could lead people to feel anxious after the communication of genetic results, but this link was not so incisive in several studies (Green et al., 2009). Furthermore, many studies concerning the emotional reactions after results receipt addressed hypothetical and non‐real situations, enrolling participants who never underwent a genetic test, or the authors never considered anxiety as an aspect that can "move" the behavioral changes after results (Oliveri, Howard, Renzi, Hansson, & Pravettoni, 2016).

There are many psycho‐cognitive factors that might affect emotional resilience to genetic bad news, and the significance people give to a genetic result (Oliveri & Pravettoni, 2018). The aim of this study was to investigate such psycho‐cognitive factors implied in genetic testing uptake, by characterizing a sample of GT consumers in the Italian context, which is still unexplored. In particular, in the following study we investigated:

1. Individual tendencies (health orientation), optimistic bias, overconfidence, risk tolerance, and regret tendencies of GT consumers;
2. Differences in risk tolerance and orientation toward health based on the personal disease history (i.e., current or past diseases; family history of a disease or genetic predisposition) and based on the type of genetic testing performed.

## 2 MATERIALS AND METHODS

### 2.1 Ethical compliance

The *Institutional Review Board at the University of Milan* (the principal coordination center of the survey), and the *Centre for Research Ethics and Bioethics, University of Uppsala* (coordinator of the *Mind the Risk* project; see funding declaration) approved this research and the study was conducted according to the Helsinki declaration.

### 2.2 Participants

The health‐care system in Italy is public and free for all citizens. Nevertheless, the regulation plan for the financial coverage of genetic tests for the population is still ongoing (Ministero della Salute, 31 January 2018). Genetic testing for disease susceptibility (e.g., analysis of BRCA mutation for breast and ovarian cancer) or for a confirmatory diagnosis (e.g., Huntington or Alzheimer diseases) is supported by the fulfillment of specific criteria, such as familiarity, young age, past history of the disease, or full‐blown symptoms. Some tests can, therefore, be directly prescribed by the doctor, and performed in the hospital or through private laboratories. However, each Italian citizen is free to purchase a panel of genetic tests for specific clinical conditions directly from private genetic laboratories. Our sample of participants was recruited from Italian citizens who purchased genetic tests directly from a private lab, with or without suggestion/prescription from a physician.

In particular, 473 clients who underwent genetic testing at *GenomaLab‐Molecular Genetics Laboratory* (an Italian private genetic laboratory located in Milan and Rome that provides several panels of genetic analysis for risk and disease susceptibility) were invited to take part in our research project, from February 2016 to September 2018. A total of 152 Italian adults accepted to answer the survey (response rate: 32%). All the clients were invited to sign an informed consent for their participation and were informed that all the responses would remain strictly confidential and anonymity was protected by the use of alphanumeric codes. Eighty‐two percent were female (*n* = 125; male: *n* = 27) and participants’ age was from 18 to 76 years old (*M*_age_ = 42.75, *SD* = 12.89). Participants were predominantly well‐educated (university degree or post‐university degree: 51.3% tot) and white‐collar (42.1%) or self‐employed people (27.6%). It is possible that the well‐educated middle class in Italy requests genetic tests in private laboratories more than the other categories since they have had higher literacy and higher economic resources.

Consumers included in this study underwent different types of genetic tests: the majority of participants underwent genetic testing for food intolerance (Nutrigenomics testing or Celiac disease testing), infertility problems (Thrombophilia testing or Celiac disease testing), cancer susceptibility (Breast and Ovarian Cancer BRCA testing or OncoNext panel for different cancers risk), and other specific tests. See Table 1 for all the types of genetic tests required by the clients.

**TABLE 1:** Type of genetic test performed by participants and main reasons for testing

Main reasons for testing	All types of genetic testing performed	Percentage of clients
Food intolerance	Nutrigenomics	33
Reproduction/infertility	Celiac disease	11
Cancer susceptibility	Thrombophilia	26
	BRCA	21
	OncoNext	3
	Huntington	2
	Hemochromatosis	1
	Fragile X	1
	Cystic fibrosis	1
	Alzheimer	1

Participants reported different personal histories of diseases: 34% of them declared that, in the *past*, they have had a relevant disease (*n* = 51), whereas 35% reported a *current disease* (*n* = 53). The majority of participants (69.9%, *n* = 94) also highlighted a *family history of diseases*, whereas 24% of them recognized that a *genetic risk predisposition* runs in the family (*n* = 37). All of the participants’ characteristics are summarized in Table 2.

**TABLE 2:** Sociodemographic characteristics of participants

Descriptive data	*n*	%
Gender		
Women	125	82.2
Men	27	17.8
Age range		
18–34	41	27.0
35–41	36	23.7
42–51	39	25.67
52–76	36	23.7
Education		
Primary school	2	0
Secondary school	7	5.9
High school	65	42.8
University degree	62	40.8
Post‐university degree	16	10.5
Occupation		
Unemployed	43	28.3
Blue‐collar	3	2.0
White‐collar	64	42.1
Self‐employed	42	27.6

### 2.3 Procedure

All the clients who underwent genetic testing for health‐related issues and predisposition to specific diseases were invited to participate in this study. However, clients who required genetic testing during pregnancy or for Medically Assisted Procreation (MAP) (e.g., PrenatalSafe, PrenatalScreen, Preimplantation genetic diagnosis) were excluded since belonging to an area accountable for being treated in a dedicated study. Those who agreed to participate completed a paper‐pencil questionnaire during their stay in the waiting room at the laboratory before giving the blood sample.

### 2.4 Measures

The following scales and questionnaires have been administered. See the Supplementary Material for a detailed description of the survey.

#### 2.4.1 Health Orientation Scale (HOS) Italian version

The HOS is a 50‐item self‐report questionnaire developed by Snell, Johnson, Lloyd, and Hoover (1991) that assesses individual tendencies associated with health (Snell et al., 1991). The scale was validated in the Italian context by Masiero et al. (2020); the Italian version composed of 36 items, grouped into seven dimensions and evaluated on a 5‐point Likert scale (from 0 “*Not at all characteristic of me*” to 4 “*Very characteristic of me*”) (Masiero et al., 2020). The score for each subscale was the sum of the values attributed to every single item (same procedure as the HOS scale from Snell and colleagues). The seven dimensions are the following:

‐ *Motivation for health promotion and prevention* (*MHPP*; 9‐items, items 4, 11, 12, 19, 20, 23, 24, 25, and 33; *α* = .882) that is referred to a high predisposition to act in favor of well‐being and to avoid risk behaviors (score ranging from 0 to 36);
‐ *Health esteem* (*HES*; 7‐items, items 3, 5, 6, 10, 14, 15, and 31; *α* = .838) describing positive thinking and confidence in handling health status, being optimistic about the future, and perceiving oneself in good physical shape (score ranging from 0 to 32);
‐ *Health image concern* (*HIC*; 5‐items, items 1, 8, 17, 22, and 30; *α* = .832) that is referred to the worry about the social impression of one's own health status (score ranging from 0 to 20);
‐ *Personal health consciousness* (*PHC*; 4‐items, items 7, 16, 21, and 29; *α* = .822) that is referred to people's tendency to think and reflect about their health, and to care about their physical status (score ranging from 0 to 16);
‐ *Health locus of control* (*HLC*; 5‐items, items 13, 26, 27, 34, and 35; *α* = .770) referred to people's belief that their health status is under their responsibility and control (score ranging from 0 to 20);
‐ *Health anxiety* (*HA*; 4‐items, items 2, 9, 18, and 31; *α* = .797) which refers to mood factors, such as worry and anxiety, that modulate individuals’ health perception (score ranging from 0 to 16);
‐ *Health expectations* (*HEX*; 2‐items, 28 and 36; *α* = .716) referred to negative expectations of one's own future health status (score ranging from 0 to 8).

#### 2.4.2 Optimistic bias Weinstein and Klein (2015)

Six questions have been used to assess participants’ optimistic bias, related to their perceived risk of incurring in negative events. Optimistic bias refers to the belief that your own chances of experiencing negative events are lower than the one of your peers. Our approach consisted in asking participants to make two kinds of judgments—an estimate of their own risk (on a 10‐point likelihood scale, from 1% to 100%) and an estimate of the risk of the average peers (on a 10‐point likelihood scale, from 1% to 100%) of incurring in negative events. Optimistic bias was computed subtracting the own risk from peers’ risk. If the difference was not zero, a bias could be said to exist (*negative scores* = pessimistic bias; *positive scores* = optimistic bias).

#### 2.4.3 Overconfidence

We administered to the participants the following question retrieved from the contribution of Pan and Stateman (Pan & Statman, 2012). The authors created the following item (evaluated on a 10‐point Likert scale) to assess overconfidence in taking financial risks: *Some people believe that they can pick stocks that would earn higher‐than‐average returns. Other people believe that they are unable to do so. Please indicate your belief by circling the number on a scale ranging from “Strongly believe I cannot pick higher‐than‐average stocks” to “Strongly believe I can pick higher‐than‐average stocks*” (p. 59). Our aim was to evaluate participants’ overall risk‐taking tendency and make a parallelism with their propensity in taking risks concerning their health. High scores indicate participants’ higher confidence in taking very high risks.

#### 2.4.4 Regret scale

The Regret Scale is a 5‐item self‐report scale designed to assess how individuals deal with situations after the decision has been made (Schwartz et al., 2002). In the present study, only two items have been considered: “*Whenever I make a choice, I’m curious about what would have happened if I had chosen differently*” and “*Once I make a decision, I don't look back*” (reverse scored) evaluated on a Likert scale from 1 (Completely Disagree) to 7 (Completely Agree). The total score has been computed summing the values of the two items.

#### 2.4.5 Health and Retirement Study (HRS)

The Health and Retirement Study (1997) explores the dimension of risk tolerance, which is the amount of risk and uncertainty that someone is able to handle in making a decision (Barsky, Juster, Kimball, & Shapiro, 1997). It uses the following scenario: “*Suppose that you are the only income earner in the family, and you have a good job guaranteed to give you your current (family) income every year for life. You are given the opportunity to take a new and equally good job, with a 50–50 chance it will double your (family) income and a 50–50 chance that it will cut your (family) income by a third. Would you take the new job?* Individuals accepting this new, risky job then consider one with a higher downside risk: *Suppose the chances were 50–50 that it would double your (family) income, and 50–50 that it would cut it in half. Would you still take the new job?* Those initially declining the new job consider one with a lower downside risk: *Suppose the chances were 50–50 that it would double your (family) income and 50–50 that it would cut it by 20 percent. Would you then take the new job?* These two responses order individuals in four categories: *unwilling to risk a one‐fifth income cut* [Very low‐risk tolerance], *willing to risk at most a one‐third cut* [Low‐risk tolerance], *willing to risk a one‐third to a one‐half cut* [Medium‐risk tolerance], and *willing to risk at least a one‐half cut* [High‐risk tolerance] (p. 2).

#### 2.4.6 Disease‐related history

The clients were invited to indicate if they have had a *past history of illness*, *current diseases*, or *familial diseases* through Yes or No answers. We also explored the consumers' awareness about the probable genetic basis of the disease history they reported, through a multiple choice answer: Yes, No, I do not know.

The overall time required to fill in the survey was 20 min.

### 2.5 Data analysis

In order to explore the overall individual tendencies of participants (health orientation, optimistic bias, overconfidence, risk tolerance, and regret), we performed descriptive analysis (frequencies and/or mean and standard deviation scores).

Chi‐square tests were performed to make comparisons in risk tolerance among groups, distinguished on the basis of clients’ personal history of disease and the type of genetic testing they asked for. Expected values and residuals in every box were calculated, in order to verify if a specific group gave a significantly higher or lower rate of response (observed values) to certain items, compared to the percentage expected and calculated on the number of subjects recruited. In the interpretation of the standardized residuals, 1.96 was considered to be the discriminant value for a confidence level of 95%. Independent sample *t*‐test and one‐way analysis of variance (ANOVA) were also computed in order to assess the possible differences in health orientation on the basis of the personal history of disease (*past*, *current*, *family history*, or *genetic* risks) and the type of genetic testing performed by the clients. The LSD post hoc test was used to determine which groups were significantly different.

Analyses were performed with the statistical software analysis package *SPSS* (Version 20.0).

## 3 RESULTS

### 3.1 Individual tendencies (health orientation), optimistic bias, overconfidence, risk tolerance, and regret tendencies of GT consumers

**FIGURE 1:**
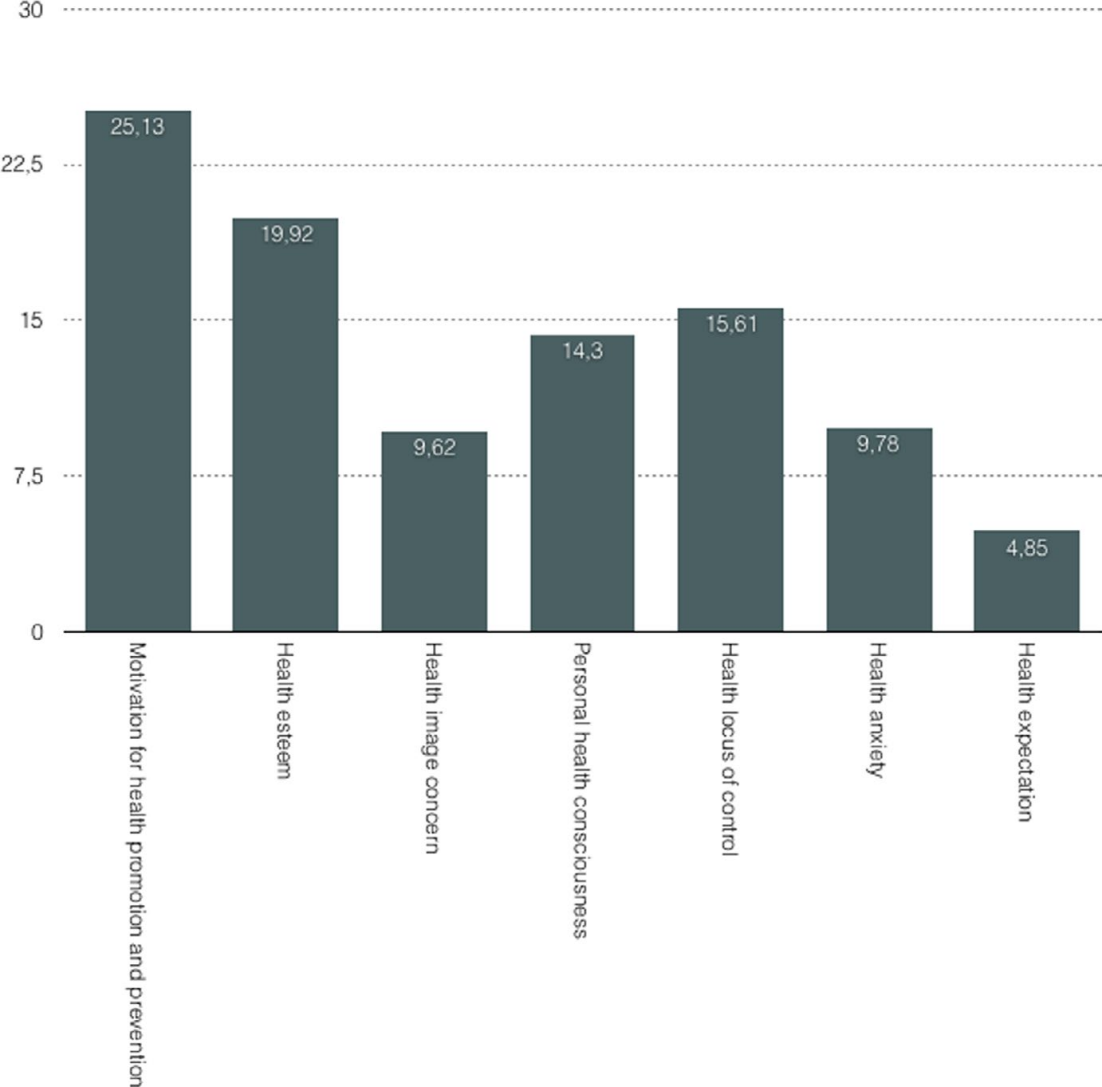
Clients’ mean score for each factor of the HOS Italian version

Participants showed a high motivation to improve and preserve their well‐being, adopting preventive behaviors (*MHPP*; *M* = 25.13 over maximum score of 36, *SD* = 4.85); showed high confidence in handling their health status, perceiving themselves as being in good physical shape (*HES*; *M* = 19.92 over a maximum score of 32, *SD* = 3.95), and showed a tendency to believe that their health is under their responsibility (*HLC*; *M* = 15.61 over a maximum score of 20, *SD* = 2.85). Consumers also showed to be strongly aware of their physical status (*PHC*; *M* = 14.30 over a maximum score of 16, *SD* = 2.85). See Figure 1 for a detailed description.

**FIGURE 2:**
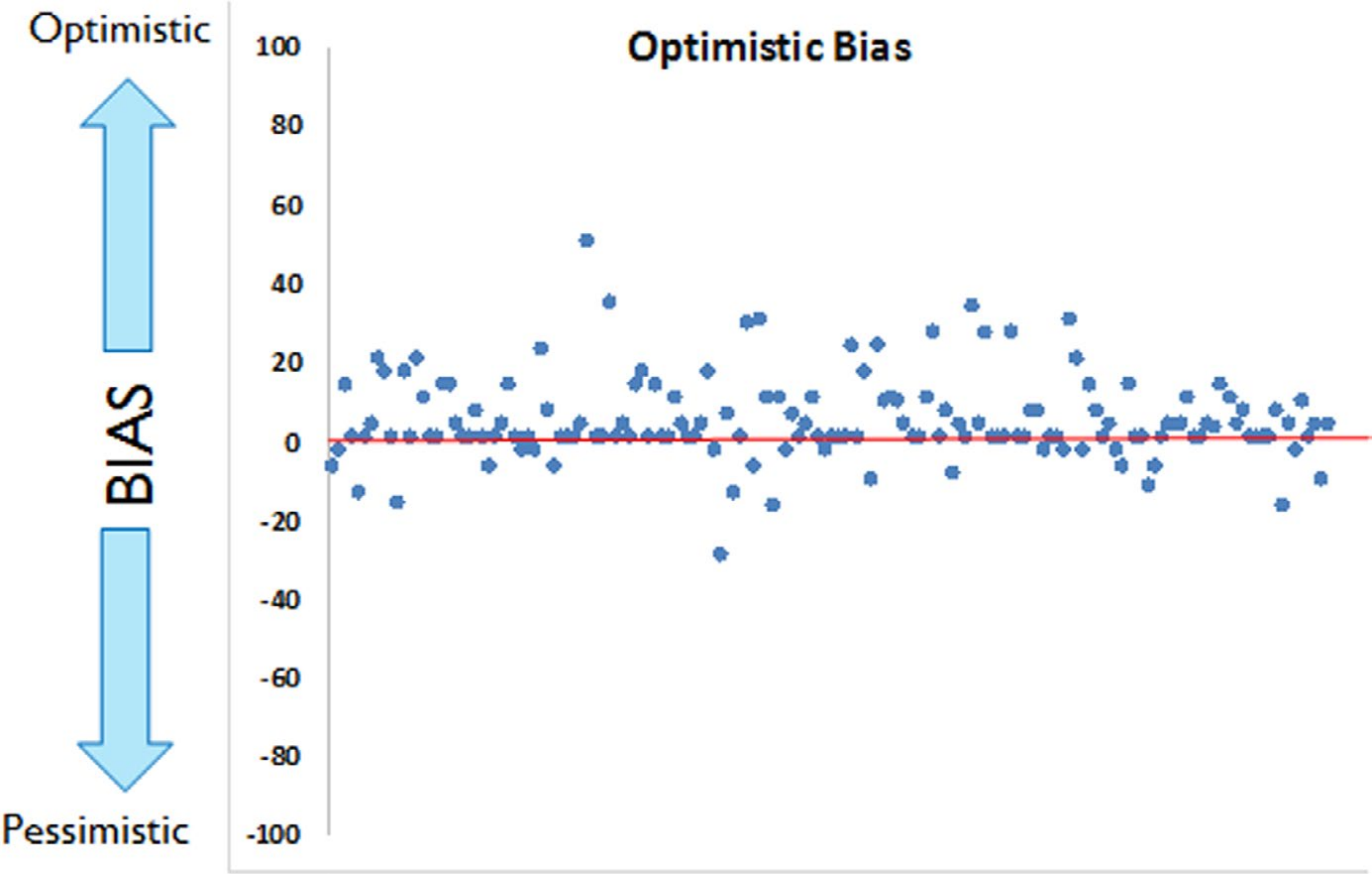
Participants' distribution for the Optimistic Bias

Additionally, data showed that our sample of Italian consumers does not have high overconfidence about their ability in taking risks (*M* = 2.81, *SD* = 2.00) and do not have an optimistic bias. As reported in Figure 2, the majority of participants scored around 0 with a slight tendency of consumers toward a “safe” optimism. Results suggested that consumers believe that their chances of experiencing negative events (such as a specific disease) are the same (or slightly lower) than those of their peers.

Considering the amount of risk and uncertainty that our sample of Italian GT consumers seem to be able to handle (risk tolerance), data showed that around half of the participants had an overall low‐risk tolerance (36.7% very low‐risk tolerance and 15.1% low‐risk tolerance) and the other half ranged from medium‐ to high‐risk tolerance (27.3% medium risk tolerance and 20.9% high‐risk tolerance).

Lastly, participants had no regret tendencies (*M* = 5.58; *SD* = 1.86), showing no personal disposition to look back after having made a decision and think about what would have happened if they had chosen differently.

### 3.2 Differences in risk tolerance and health orientation based on the personal history of disease and genetic testing performed

Chi‐square statistics showed that participants who have had a *past* disease tended to be significantly less tolerant to risk, compared to consumers who did not have a past disease (*χ^2^* (3,138) = 9.439, *p* < .024). Whereas participants with *current* disease (*χ^2^* (3,139) = 5.780, *p* = .123), *familial* (*χ^2^* (3,124) = 3.837, *p* = .280), or *genetic* history of disease in the family (*χ^2^* (6,139) = 5.329, *p* = .502) did not show statistical significant differences in the level of risk tolerance.

**FIGURE 3:**
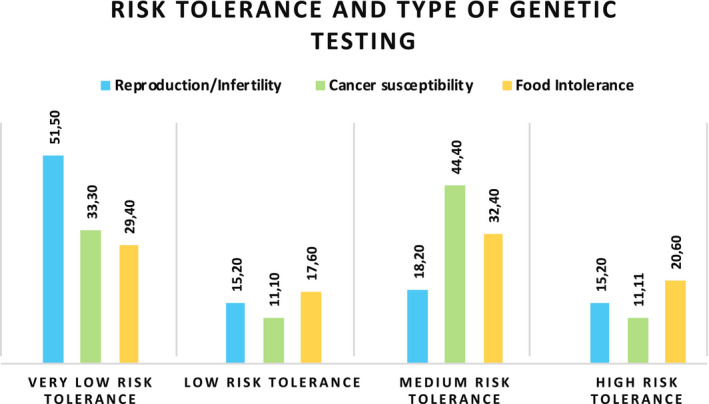
Level of risk tolerance on the basis of the type of genetic testing

The level of risk tolerance did not differ on the basis of the type of genetic testing (cancer susceptibility, reproduction/infertility, food intolerance) that clients underwent (*χ^2^*(6) = 6.211, *p* = .400). Nevertheless, the analysis of standardized residuals shows that people who underwent genetic testing for reproductive/infertility reasons (51.5% [*n* = 17]) had a close to significant lower risk tolerance, compared to the other groups (*cancer* 33.3% [*n* = 6] vs *food intolerance* 29.4% [*n* = 10]; standardized residual = 1.9; Figure 3).

Independent sample *t*‐test revealed that participants with *family* or *past* history of diseases did not differ in their orientation toward health. On the contrary, participants with *current* disease tended to show less health esteem (*HES; t*(140) = −3.09, *p* = .002; *M* = 18.38, *SD* = 4.76) and to have more negative expectations for their future health status (*HEX; t*(143) = 2.765, *p* = .006; *M* = 5.44, *SD* = 1.92) compared to consumers without current disease (*M* = 20.70, *SD* = 3.95; *M* = 4.54, *SD* = 1.84, respectively).

Participants who are aware to have a *genetic* disease in the family significantly differed in the motivation for health promotion (*MHPP*; ANOVA: *F*(2)5.85, *p* = .004, *η^2^* = 0.078) and in the expectations for their future health status (*HEX;* ANOVA: *F*(2)3.37, *p* = .037, *η^2^* = 0.045) compared to others consumers. In particular, the LSD post hoc test showed that consumers with a genetic disease in the family had lower motivation for health promotion (*p* = .026; *M* = 24.52; *SD* = 5.03) compared to those who had not (*M* = 26.81; *SD* = 4.61) or who did not know (*p* = .001; *M* = 23.80; *SD* = 4.53). In the same line, consumers with a genetic disease in the family had negative expectations for future health status (*p* = .030; *M* = 5.23; *SD* = 1.94) compared with those without a genetic disease in the family (*M* = 4.34; *SD* = 1.79) or those that did not know (*p* = .029; *M* = 5.13; *SD* = 1.93).

## 4 DISCUSSION

GT are transforming the health‐care system, the personalized medicine, and the health‐care market. In this framework is paramount to profile people who decide to undergo GT, and to understand if and how individual tendencies could influence the way people react and manage genetic risk information.

The first interesting aspect of our results is that Italian consumers seem to have a very high motivation to avoid risk conditions that could affect their health (*Motivation for health promotion and prevention)*, that they usually deeply reflect about their health status, and that they feel aware about it (*Personal health consciousness*). Moreover, they have an overall positive feeling and confidence in handling their health status (*Health esteem)*, and finally, they believe they can influence their health status through their choices (*Health locus of control*).

We asked our participants to estimate their overconfidence in taking risks and their pessimistic/optimistic tendencies toward negative situations and their health in general. They described themselves as people who usually do not feel confident in taking very high risks and who are nor optimistic or pessimistic concerning chances of negative life events (such as a disease onset) that can occur. The majority of them did not report to have regret tendencies, so they usually do not look back after having made a health‐related decision. Nothing interesting emerged concerning the level of risk tolerance in our sample of consumers, who were basically split in half about their overall tendency to tolerate risk and (uncertainty for future disease in this case, since genetic testing provides probabilistic analysis of risk).

Our results depict Italian GT consumers as a population that already have a health‐oriented lifestyle, who intentionally focus their choices for health prevention, who do not like to take risks and therefore prefer not to “bet” on their health, and probably their intention (and belief) is to acquire more health‐related information through genetic testing in order to reduce uncertainty about their current status or predisposition to disease. Their choice to undergo genetic testing does not seem to stem from a pessimistic perception of their condition, that is from the fear of having a greater chance of getting sick, but from the desire to acquire a specific type of health information which can give them a greater decision‐making “power” on how to face their current clinical condition or future risk, or promote their health in general. Indeed, predictive genetic tests are often introduced, both in clinical and general market settings, in terms of possibility to know something of your future health and counteract potential threats, contributing to a paradigm shift in which you no longer get a disease by chance but you can do something by predicting the future with GT and by implementing appropriate behaviors (Oliveri et al., 2019). Private genetic companies have always promoted their services as a way of controlling risk (e.g., online video advertisements from 23 and Me). A strong emphasis is laid on the identification of risks to one's health and life, and the possible ways to act to diminish or face those risks. The goal is, therefore, to transform the future of one's health from a realm of uncertainty into something that can be managed (Boenink & van der Burg, 2010; Kalokairinou, Borry, & Howard, 2017; Pakholok, 2013). We could say that current debates concerning health tend to put health prevention and promotion as a great value in its temporal sense (Boenink & van der Burg, 2010).

Thus, compared to the considerations reported in the introduction, our results confirmed that the Italian genetic test users are not pessimistic or worried about their current condition but, on the contrary, they do not tolerate to take high risks and leave health to fate, they invest in their own health and consider genetic tests as a valid information to take important preventive decisions.

Life experiences and family history can play a huge role in handling with genetic risk information and their implications. We investigated whether the condition of being suffering from a clinical condition at the moment of GT testing or having suffered in the past, and/or having experienced a disease in the family (e.g., the illness of a loved one until his/her loss, or the awareness that a genetic condition runs in the family) could influence consumers’ risk tolerance, orientation toward health and toward genetic risk information. Our results showed that consumers who have had past experience with a serious disease or a clinical condition that affects well‐being are less tolerant to the risk of incurring future diseases compared to consumers who did not have such experience. Despite the level of risk tolerance does not seem to be significantly linked to the type of clinical condition investigated (serious disease such as cancer, or food intolerance, or infertility), consumers who underwent genetic testing for infertility problems showed a tendency to be less tolerant to the risk of occurring future health‐related negative events.

Not surprisingly, people who reported to have a disease at the moment of testing showed a lower tendency to “think positive” about their health and expect to be less “healthy” in the future; the same was for consumers who were already aware of a genetic predisposition in the family, who showed negative expectations for their future health and less motivation to promote it. It seems that the awareness of a genetic family‐related predisposition it is enough to generate the future expectation of pathology or clinical condition. In literature, the deterministic perception of genetic risk information has been widely discussed (Gericke et al., 2017), so the expectation toward genetic data is that it constitutes a sort of "destiny" toward a specific condition. Overall, other evidence shows that people feel confident in managing their risk but they also tend to believe that lifestyle could be useless to face their genetic predisposition and they need other more “concrete” solutions such as drug therapies or surgical interventions (Marteau et al., 2004). Consumers who already have a disease at the moment of genetic testing tend to be more “negative” concerning their health. Nevertheless, literature (Hietaranta‐Luoma, Tahvonen, Iso‐Touru, Puolijoki, & Hopia, 2014; Marteau et al., 2004) reported that people who already have physical symptoms tend to be more prone to change their lifestyle, and Oliveri et al. (2018) in their review observed that people with full‐blown symptoms might be more motivated to gather all possible health‐related information, including genetic risk information, in order to manage their risk of developing the disease (Oliveri et al., 2018).

In conclusion, our sample of clients approaching genetic testing through a private laboratory in Italy is aware, responsible, and motivated toward their health, they want to reduce uncertainty for future risks, and believe GT can help them to take steps in these directions. It is also wise to acknowledge the uncertainty of what current technology can tell and improve the way how to communicate it. The results of this kind of study could contribute to partially "cluster" the population of GT users in Europe and reflect on the possibility to entail the communication of genetic risk information based on their personal profile. Further studies should be conducted considering a long‐term monitoring of the decisions taken by consumers and changes in lifestyle after the receipt of genetic test results.

## CONFLICT OF INTEREST

The authors declare that they have no competing interests.

## AUTHOR CONTRIBUTION

SO, IC, AG, and GP conceived the design of the present study and were in charge of overall direction and planning. SO, FS, and MB enrolled participants and collected data. SO, ID, IC, and CC wrote the manuscript with input from all authors. All authors discussed the results and commented on the manuscript.

## Supporting information

Supplementary Material

## Data Availability

The datasets used and/or analyzed during the current study are available from the corresponding author on reasonable request.

## References

[mgg31291-bib-0001] Achar, C. , So, J. , Agrawal, N. , & Duhachek, A. (2016). What we feel and why we buy: The influence of emotions on consumer decision‐making. Current Opinion in Psychology, 10, 166–170. 10.1016/j.copsyc.2016.01.009

[mgg31291-bib-0002] Barsky, R. B. , Juster, F. T. , Kimball, M. S. , & Shapiro, M. D. (1997). Preference parameters and behavioral heterogeneity: An experimental approach in the health and retirement study. The Quarterly Journal of Economics, 112(2), 537–579. 10.1162/003355397555280

[mgg31291-bib-0003] Boenink, M. , & van der Burg, S. (2010). Informed decision making about predictive DNA tests: Arguments for more public visibility of personal deliberations about the good life. Medicine, Health Care and Philosophy, 13(2), 127–138. 10.1007/s11019-009-9227-6 PMC2848344 19876767

[mgg31291-bib-0004] Collins, J. , Ryan, L. , & Truby, H. (2014). A systematic review of the factors associated with interest in predictive genetic testing for obesity, type II diabetes and heart disease. Journal of Human Nutrition and Dietetics, 27(5), 479–488. 10.1111/jhn.12179 24236642

[mgg31291-bib-0005] Crow, D. (2019). A new wave of genomics for all. Cell, 177(1), 5–7. 10.1016/j.cell.2019.02.041 30901548

[mgg31291-bib-0006] Fallaize, R. , Macready, A. L. , Butler, L. T. , Ellis, J. A. , & Lovegrove, J. A. (2013). An insight into the public acceptance of nutrigenomic‐based personalised nutrition. Nutrition Research Reviews, 26(1), 39–48. 10.1017/S0954422413000024 23561449

[mgg31291-bib-0007] Gericke, N. , Carver, R. , Castéra, J. , Evangelista, N. A. M. , Marre, C. C. , & El‐Hani, C. N. (2017). Exploring relationships among belief in genetic determinism, genetics knowledge, and social factors. Science & Education, 26(10), 1223–1259. 10.1007/s11191-017-9950-y

[mgg31291-bib-0008] Green, R. C. , Roberts, J. S. , Cupples, L. A. , Relkin, N. R. , Whitehouse, P. J. , Brown, T. , … Farrer, L. A. (2009). Disclosure of APOE genotype for risk of Alzheimer’s disease. New England Journal of Medicine, 361(3), 245–254. 10.1056/NEJMoa0809578 19605829 PMC2778270

[mgg31291-bib-0009] Grishin, D. , Obbad, K. , Estep, P. , Quinn, K. , Zaranek, S. W. , Zaranek, A. W. , … Church, G. (2018). Accelerating genomic data generation and facilitating genomic data access using decentralization, privacy‐preserving technologies and equitable compensation. Blockchain in Healthcare Today, 1, 1–23. 10.30953/bhty.v1.34

[mgg31291-bib-0010] Hietaranta‐Luoma, H.‐L. , Tahvonen, R. , Iso‐Touru, T. , Puolijoki, H. , & Hopia, A. (2014). An intervention study of individual, apoE genotype‐based dietary and physical‐activity advice: Impact on health behavior. Journal of Nutrigenetics and Nutrigenomics, 7(3), 161–174. 10.1159/000371743 25720616

[mgg31291-bib-0011] Jamal, L. , Robinson, J. O. , Christensen, K. D. , Blumenthal‐Barby, J. , Slashinski, M. J. , Perry, D. L. , … McGuire, A. L. (2017). When bins blur: Patient perspectives on categories of results from clinical whole genome sequencing. AJOB Empirical Bioethics, 8(2), 82–88. 10.1080/23294515.2017.1287786 28949844 PMC6647021

[mgg31291-bib-0012] Kalokairinou, L. , Borry, P. , & Howard, H. C. (2017). Regulating the advertising of genetic tests in Europe: A balancing act. Journal of Medical Genetics, 54(10), 651–656. 10.1136/jmedgenet-2017-104531 28735297

[mgg31291-bib-0013] Marteau, T. , Senior, V. , Humphries, S. E. , Bobrow, M. , Cranston, T. , Crook, M. A. , … Wray, R. (2004). Psychological impact of genetic testing for familial hypercholesterolemia within a previously aware population: A randomized controlled trial. American Journal of Medical Genetics, 128A(3), 285–293. 10.1002/ajmg.a.30102 15216550

[mgg31291-bib-0014] Masiero, M. , Oliveri, S. , Cutica, I. , Monzani, D. , Faccio, F. , Mazzocco, K. , & Pravettoni, G. (2020). The psychometric properties of the Italian adaptation of the Health Orientation Scale (HOS). Health and Quality of Life Outcomes, 18(1), 69 10.1186/s12955-020-01298-z 32169082 PMC7071689

[mgg31291-bib-0015] Oliveri, S. , Ferrari, F. , Manfrinati, A. , & Pravettoni, G. (2018). A systematic review of the psychological implications of genetic testing: A comparative analysis among cardiovascular, neurodegenerative and cancer diseases. Frontiers in Genetics, 10(9), 624 10.3389/fgene.2018.00624 PMC6295518 30619456

[mgg31291-bib-0016] Oliveri, S. , Howard, H. C. , Renzi, C. , Hansson, M. G. , & Pravettoni, G. (2016). Anxiety delivered direct‐to‐consumer: Are we asking the right questions about the impacts of DTC genetic testing? Journal of Medical Genetics, 53(12), 798–799. 10.1136/jmedgenet-2016-104184 27647845

[mgg31291-bib-0017] Oliveri, S. , & Pravettoni, G. (2016). The disclosure of direct‐to‐consumer genetic testing: Sounding out the psychological perspective of consumers. Biology and Medicine, 8(5), 1–4. 10.4172/0974-8369.1000316

[mgg31291-bib-0018] Oliveri, S. , & Pravettoni, G. (2018). Capturing how individuals perceive genetic risk information: A phenomenological perspective. Journal of Risk Research, 21(2), 259–267. 10.1080/13669877.2017.1281333

[mgg31291-bib-0019] Oliveri, S. , Pravettoni, G. , Fioretti, C. , & Hansson, M. G. (2016). Let the individuals directly concerned decide: A solution to tragic choices in genetic risk information. Public Health Genomics, 19(5), 307–313. 10.1159/000448913 27603671

[mgg31291-bib-0020] Oliveri, S. , Renzi, C. , Masiero, M. , & Pravettoni, G. (2015). Living at risk: Factors that affect the experience of direct‐to‐consumer genetic testing. Mayo Clinic Proceedings, 90(10), 1323–1326. 10.1016/j.mayocp.2015.06.014 26434959

[mgg31291-bib-0021] Oliveri, S. , Scotto, L. , Ongaro, G. , Triberti, S. , Guiddi, P. , & Pravettoni, G. (2019). You do not get cancer by chance”: Communicating the role of environmental causes in cancer diseases and the risk of a “guilt rhetoric. Psycho‐Oncology, 28(12), 2422–2424. 10.1002/pon.5224 31512349 PMC6916158

[mgg31291-bib-0022] Pakholok, O. (2013). The idea of healthy lifestyle and its transformation into health‐oriented lifestyle in contemporary society. SAGE Open, 3(3), 215824401350028 10.1177/2158244013500281

[mgg31291-bib-0023] Pan, C. H. , & Statman, M. (2012). Questionnaires of risk tolerance, regret, overconfidence, and other investor propensities. SSRN Electronic Journal, 13(1), 54–63. 10.2139/ssrn.1549912

[mgg31291-bib-0024] Schwartz, B. , Ward, A. , Monterosso, J. , Lyubomirsky, S. , White, K. , & Lehman, D. R. (2002). Maximizing versus satisficing: Happiness is a matter of choice. Journal of Personality and Social Psychology, 83(5), 1178–1197. 10.1037/0022-3514.83.5.1178 12416921

[mgg31291-bib-0025] Snell, W. E. , Johnson, G. , Lloyd, P. J. , & Hoover, M. W. (1991). The Health Orientation Scale: A measure of psychological tendencies associated with health. European Journal of Personality, 5(2), 169–183. 10.1002/per.2410050208

[mgg31291-bib-0026] Stewart‐Knox, B. J. , Bunting, B. P. , Gilpin, S. , Parr, H. J. , Pinhão, S. , Strain, J. J. , … Gibney, M. (2008). Attitudes toward genetic testing and personalised nutrition in a representative sample of European consumers. British Journal of Nutrition, 101(7), 982–989. 10.1017/S0007114508055657 18775102

[mgg31291-bib-0027] Weinstein, N. D. , & Klein, W. M. P. (2015). Health risk appraisal and optimistic bias In Wright J. D. (Ed.), International encyclopedia of the social & behavioral sciences, (2nd ed, pp. 698–701). Oxford, UK: Elsevier 10.1016/B978-0-08-097086-8.25012-5

