# Supplementary material for: Health orientation and individual tendencies of a sample of Italian genetic testing consumers
Source: Mol Genet Genomic Med. 2020 Jun 5;8(8):e1291. doi: 10.1002/mgg3.1291 (PMC7434739; doi:10.1002/mgg3.1291)
Supplement: Supplementary file 1 — Supplementary Material [file MGG3-8-e1291-s001.docx]

Genetic testing Users Questionnaire

1. Write your birth date in the following alphanumeric format (e.g. 15_06_1976_G)

_________________________________________

2. Insert your Genoma code^[[1]](#footnote-1)^

________________________________

3. Are you male or female?

- Male
- Female

4. What is your age?

________________________

5. What is the highest degree or level of education you have completed?

- Primary school
- Secondary school
- High school diploma or equivalent
- University degree (Bachelor’s or Master’s degree)
- Post University degree (Ph.D. or specialization)
- Other (please specify)

______________________________

6. Which country do you come from?

_______________________

7. Are you currently…?

- Unemployed (Student, Retired, Homemaker, Unable to work, looking for work, Out of work but not currently looking for work)
- Blue collar
- White collar
- Self Employed
- Other (please specify)

____________________________________

8. What is the reason why you decided to undergo a genetic analysis?

__________________________________

9. Have you suffered for specific illnesses in the past? If Yes please specify.

- No
- Yes

Please specify

______________________________

10. Do you currently suffer for specific illnesses? If Yes, please specify.

- No
- Yes

Please specify

______________________________

11. In your family health-span history, is there a relevant experience of illness? If yes, please specify which one.

- No
- I don't know
- Yes

Please specify which illness

_____________________________

12. Do you have a family history of genetically/inherited diseases?

- No, I have not
- I don't know
- Yes, I have

If yes, please specify

_____________________________

Please, express your opinion about the following questions. Put a cross on your answer.

13. How likely is that a person of your age and country could be involved in a traffic accident caused by other people, in the course of his/her life?

1% 10% 20% 30% 40% 50% 60% 70% 80% 90% 100%

14. How likely is that a person of your age and country could undergo surgery in the next 10 years?

1% 10% 20% 30% 40% 50% 60% 70% 80% 90% 100%

15. How likely is it that the financial investments of a person of your age and country will double in the next five years?

1% 10% 20% 30% 40% 50% 60% 70% 80% 90% 100%

16. Some people believe that they can pick stocks that would earn higher-than-average returns. Other people believe that they are unable to do so. Please indicate your belief by signing the number on a scale ranging from “Strongly believe I cannot pick higher-than-average stocks” to “Strongly believe I can pick higher-than-average stocks.” Scores range from 1 to 10 where high numbers indicate a belief that one is able to pick higher-than-average stocks.


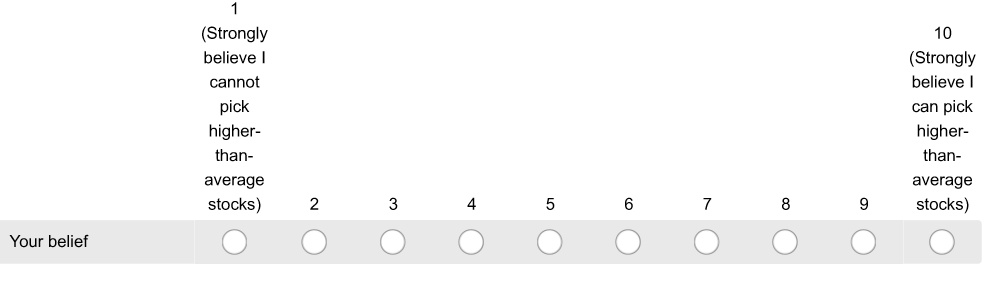


17. The items listed below refer to people's health. Please read each item carefully and decide to what extent it is typical of you. Give each item a rating of how much it applies to you by using the following scale:

*Not at all characteristic of me (0), Slightly characteristic of me (1), Somewhat characteristic of me (2), Moderately characteristic of me (3), Very characteristic of me (4).*


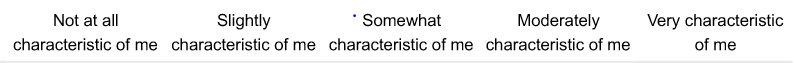


I sometimes wonder what others think of my physical health.
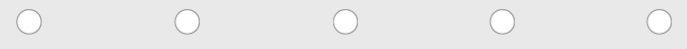


I feel anxious when I think about my health.
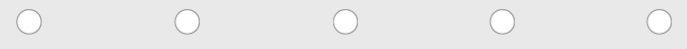


I feel confident about the status of my health.
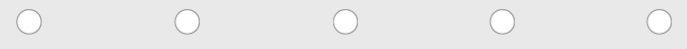


I do things that keep me from becoming physically unhealthy.
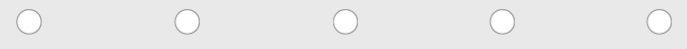


I expect that my health will be excellent in the future.
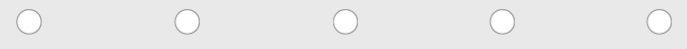


I am in good physical health.
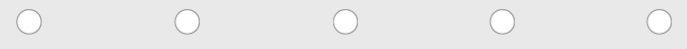


I notice immediately when my body doesn't feel healthy.
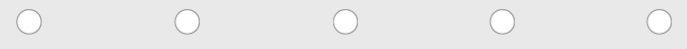


I’m very concerned with how others evaluate my

physical health.
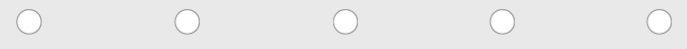


I'm worried about how healthy my body is
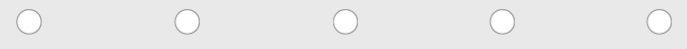


I rarely become discouraged about my health.
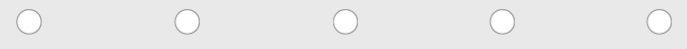


I am motivated to keep myself from becoming

physically unhealthy
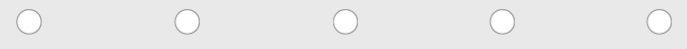


I am strongly motivated to devote time and effort

to my physical health.
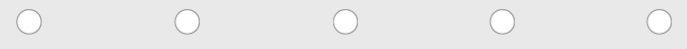


My health is something that I alone am responsible for
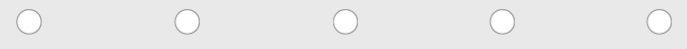


I believe that the future status of my physical health

will be positive.
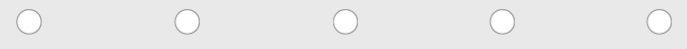


My body is in good physical shape.
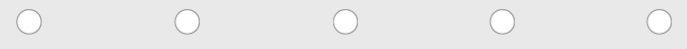


I'm sensitive to internal bodily cues about my health.
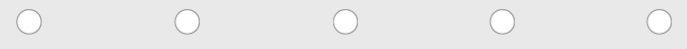


I'm very aware of what others think of my physical health.
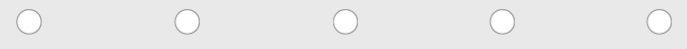


Thinking about my health leaves me with an uneasy feeling.
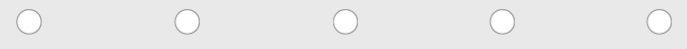


I try to avoid engaging in behaviors that undermine

my physical health.
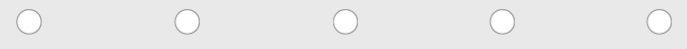


I have a strong desire to keep myself physically healthy.
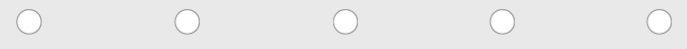


I know immediately when I'm not feeling in great health.
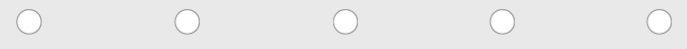


I'm concerned about how my physical health

appears to others.
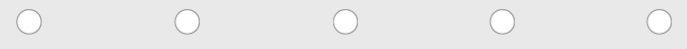


I usually worry about whether I am in good health.
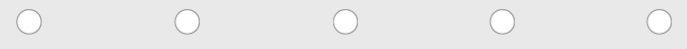


I really want to prevent myself from getting out of shape.
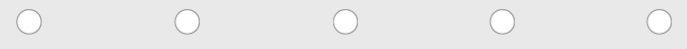


It's really important to me that I keep myself in proper

physical health.
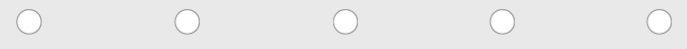


What happens to my physical health is my own doing.
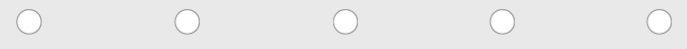


Being in excellent physical shape has little or nothing to do

with luck.
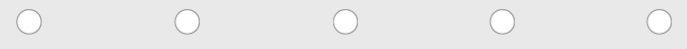


I will probably experience a number of health problems

in the future
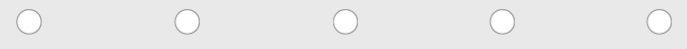


I'm very aware of changes in my physical health
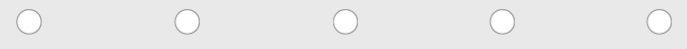


I'm concerned about what other people think of

my physical health.
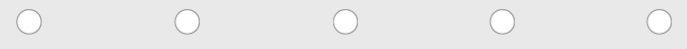


I feel nervous when I think about the status of

my physical health.
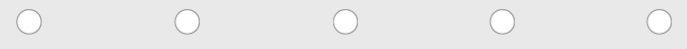


I feel that I have handled my health very well.
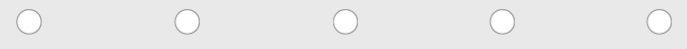


I am motivated to avoid being in terrible physical shape.
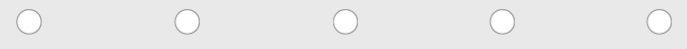


Being in good physical health is a matter of

my own ability and effort.
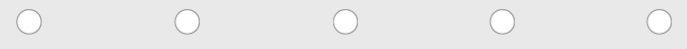


I don't believe that chance or luck play any role in

the status of my physical health
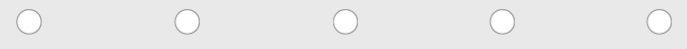


I anticipate that my physical health will deteriorate

in the future
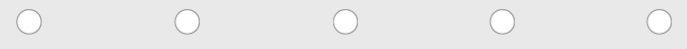


18. Please read each item carefully and decide to what extent it is typical of you. Give each item a rating of how much it applies to you by using the following scale:

*Completely Disagree (1), Mostly Disagree (2), Slightly Disagree (3), Neither agree nor disagree (4), Slightly Agree (5), Mostly Agree (6), Completely Agree (7)*

Whenever I make a choice, I’m curious about what would have happened if I had chosen differently

1 2 3 4 5 6 7


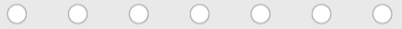


Once I make a decision, I don’t look back

1 2 3 4 5 6 7


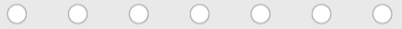


Please, express your opinion about the following questions. Put a cross on your answer.

19. How likely is it that you remain involved in a traffic accident caused by other people, in the course of your life?

1% 10% 20% 30% 40% 50% 60% 70% 80% 90% 100%

20. How likely is that you undergo surgery in the next 10 years?

1% 10% 20% 30% 40% 50% 60% 70% 80% 90% 100%

21. How likely is it that your financial investments will double over the next five years?

1% 10% 20% 30% 40% 50% 60% 70% 80% 90% 100%

22. Suppose that you are the only income earner in the family, and you have a good job guaranteed to give you your current (family) income every year for life. You are given the opportunity to take a new and equally good job, with a 50–50 chance it will double your (family) income and a 50–50 chance that it will cut your (family) income by a third. Would you take the new job?

- Yes
- No

23. If your answer to question n22 is “yes”: Suppose the chances were 50–50 that it would double your (family) income, and 50– 50 that it would cut it in half. Would you still take the new job?

- Yes
- No

24. If your answer to question n22 is “no”: Suppose the chances were 50–50 that it would double your (family) income and 50– 50 that it would cut it by 20 percent. Would you then take the new job?”

- Yes
- No

1. The Genoma code is the ID code recorded by the Lab to identify the exact panel of genetic testing performed for the client, and correlated results. [↑](#footnote-ref-1)
